# Cystathionine β‐Synthase Deficiency in the E‐HOD Registry—Part II: Dietary and Pharmacological Treatment

**DOI:** 10.1002/jimd.12844

**Published:** 2025-01-13

**Authors:** Andrew A. M. Morris, Jitka Sokolová, Markéta Pavlíková, Florian Gleich, Stefan Kölker, Carlo Dionisi‐Vici, Matthias R. Baumgartner, Luciana Hannibal, Henk J. Blom, Martina Huemer, Viktor Kožich, E‐HOD Consortium, Rodrigo R. Arantes, Francisco Arrieta Blanco, Anna Baghdasaryan, Diana Ballhausen, Javier Blasco‐Alonso, Martijn Brouwers, María Bueno, Rosa Burgos, Elvira Cañedo Villarroya, Aline Cano, María‐Luz Couce, Ellen Crushell, Javier De Las Heras, Can Ficicioglu, María Concepción García Jiménez, Ana Gaspar, Domingo González‐Lamuño Leguina, Kimberly A. Chapman, Yin‐Hsiu Chien, Mirian C. H. Janssen, Pavel Ješina, Christina Kaufman, Robin Lachmann, Christian Lavigne, Allan M. Lund, Natalia Lüsebrink, Francois Maillot, Ana Maria Martins, Silvia Meavilla Olivas, Karine Mention, Isidro Vitoria Miñana, Fanny Mochel, Ahmad Monavari, Sónia Moreira, Carolina Araujo Moreno, Helen Mundy, Elaine Murphy, Giorgia Olivieri, Stéphanie Paquay, Luís Peña‐Quintana, Danijela Petković Ramadža, Gloria Liliana Porras‐Hurtado, Pilar Quijada‐Fraile, Isabelle Redonnet‐Vernhet, Alexander Rennings, Mònica Ruiz Pons, Saikat Santra, Aude Servais, Maria Cristina Schiaffino, Manuel Schiff, Bernd C. Schwahn, Ida V. D. Schwartz, Leighann J. Sremba, Collette Stainforth, Karolina Maria Stepien, Jolanta Sykut‐Cegielska, Allyson Terry, Inmaculada Vives Piñera, Monique Williams, Jiří Zeman, Matthias Zielonka

**Affiliations:** ^1^ Manchester Centre for Genomic Medicine Manchester University Hospitals NHS Trust Manchester UK; ^2^ Department of Pediatrics and Inherited Metabolic Disorders Charles University‐First Faculty of Medicine and General University Hospital in Prague Prague Czechia; ^3^ Department of Probability and Mathematical Statistics Charles University‐Faculty of Mathematics and Physics Prague Czechia; ^4^ Centre for Pediatric and Adolescent Medicine, Department of Pediatrics I, Division of Pediatric Neurology and Metabolic Medicine Medical Faculty of Heidelberg, Heidelberg University Heidelberg Germany; ^5^ Division of Metabolism Bambino Gesù Children's Research Hospital Rome Italy; ^6^ Division of Metabolism and Children's Research Center University Children's Hospital Zurich Switzerland; ^7^ Laboratory of Clinical Biochemistry and Metabolism, Center for General Pediatrics and Adolescent Medicine, Medical Center University of Freiburg Freiburg Germany; ^8^ Department of Clinical Genetics Center for Lysosomal and Metabolic Diseases, Erasmus Medical Center Rotterdam The Netherlands; ^9^ Department of Pediatrics Landeskrankenhaus Bregenz Bregenz Austria

**Keywords:** betaine, homocystinuria, methionine, newborn screening, protein restriction, pyridoxine

## Abstract

Cystathionine β‐synthase (CBS) deficiency (classical homocystinuria) has a wide range of severity. Mildly affected patients typically present as adults with thromboembolism and respond to treatment with pyridoxine. Severely affected patients usually present during childhood with learning difficulties, ectopia lentis and skeletal abnormalities; they are pyridoxine non‐responders (NR) or partial responders (PR) and require treatment with a low‐methionine diet and/or betaine. The European network and registry for Homocystinurias and methylation Defects (E‐HOD) has published management guidelines for CBS deficiency and recommended keeping plasma total homocysteine (tHcy) concentrations below 100 μmol/L. We have now analysed data from 311 patients in the registry to see how closely treatment follows the guidelines. Pyridoxine‐responsive patients generally achieved tHcy concentrations below 50 μmol/L, but many NRs and PRs had a mean tHcy considerably above 100 μmol/L. Most NRs were managed with betaine and a special diet. This usually involved severe protein restriction and a methionine‐free amino acid mixture, but some patients had a natural protein intake substantially above the WHO safe minimum. Work is needed on the methionine content of dietary protein as estimates vary widely. Contrary to the guidelines, most NRs were on pyridoxine, sometimes at dangerously high doses. tHcy concentrations were similar in groups prescribed high or low betaine doses and natural protein intakes. High tHcy levels were probably often due to poor compliance. Comparing time‐to‐event graphs for NR patients detected by newborn screening and those ascertained clinically showed that treatment could prevent thromboembolism (risk ratio 0.073) and lens dislocation (risk ratio 0.069).

## 1 Introduction

Cystathionine β‐synthase (CBS) deficiency is a rare autosomal recessive disorder also known as classical homocystinuria (OMIM 236200). Homocysteine is a non‐structural amino acid formed during the catabolism of methionine, an essential amino acid. CBS (EC 4.2.1.22) converts homocysteine to cystathionine, which can then be converted to the non‐essential amino acid, cysteine, by γ‐cystathionase.

The main clinical features of CBS deficiency are dislocation of the optic lenses, osteoporosis and a ‘marfanoid’ habitus, learning difficulties and a predisposition to thromboembolism. The severity of the condition varies markedly [1, 2]. Some patients have a severe childhood‐onset multisystem disease, whilst others only suffer thromboses as adults or may remain asymptomatic throughout life [3]. The pathogenesis is incompletely understood [4], but hyperhomocysteinemia and cysteine deficiency contribute through direct toxicity, induction of N‐homocysteinylation of lysine residues, mitochondrial fission and altered synthesis of hydrogen sulphide and glutathione [5, 6, 7, 8].

The three main forms of treatment for CBS deficiency are pyridoxine, betaine and a low‐methionine diet (i.e., low‐protein with a methionine‐free amino acid supplement). Approximately 50% of CBS‐deficient patients respond to treatment with pyridoxine (vitamin B_6_) [1]. This is thought to act by increasing the concentration of pyridoxal 5′‐phosphate, which is a cofactor for CBS and probably acts as a chaperone for the nascent enzyme [9]. We have described a standardised test for pyridoxine responsiveness [10] and categorised patients as pyridoxine non‐responders (NRs), partial responders (PRs), whose plasma total homocysteine concentration (tHcy) falls by more than 20% on pyridoxine but remains above 50 μmol/L, full responders (FRs), whose tHcy falls below 50 μmol/L and extreme responders (ERs), whose tHcy falls below 50 μmol/L on less than 1 mg/kg/day of pyridoxine [2].

Patients who do not respond to pyridoxine (or only respond partially) are treated with a special diet and/or betaine. Betaine (*N*,*N*,*N*‐trimethylglycine) lowers homocysteine concentrations in CBS deficiency by donating a methyl group and converting it to methionine. Betaine treatment raises the methionine concentration and very high levels have been associated with cerebral oedema [11]. The standard dietary management is to restrict natural protein severely and to give a methionine‐free amino acid mixture (AAM; which usually also contains vitamins and minerals). Strict dietary management is, however, difficult and the AAMs are unpalatable. Some patients are, therefore, managed with a milder protein restriction (with or without an AAM), combined with betaine.

Newborn screening (NBS) allows CBS‐deficient patients to be treated before they suffer complications; moreover, it is easier to start dietary treatment in infants than in older individuals. NBS has been undertaken in Dublin since 1971 and is now done in a number of countries using various methods [12, 13, 14, 15]. Methionine or methionine‐to‐phenylalanine ratio has typically been the primary marker with second‐tier testing of tHcy. Unfortunately, this misses almost all pyridoxine‐responsive patients and some NRs [2, 13, 14, 15].

The European network and registry for Homocystinurias and methylation Defects (E‐HOD) started as an EU‐funded project in 2013, but it is now an international consortium involving 70 centres from all over the world. E‐HOD members have developed evidence‐based guidelines for the diagnosis and management of CBS deficiency [10]. A review of an Irish NBS‐detected cohort found no complications in patients whose lifetime median plasma free homocystine was no more than 11 μmol/L [16]. This corresponds to a plasma tHcy concentration of approximately 120 μmol/L but we recommended keeping it below 100 μmol/L.

E‐HOD has also established a web‐based registry (https://www.ehod‐registry.org/about‐ehod), with comprehensive pseudonymised data about patients' clinical and biochemical features and treatment, managed by the registry study team at Heidelberg University Hospital. Data from the registry has previously been used to review the natural history of CBS deficiency [2] and to evaluate the safety of betaine anhydrous treatment in individuals with CBS deficiency and other forms of homocystinuria [17]. In this study, we have analysed the registry data to see how closely current practice follows the published guidelines and whether the biochemical results meet the recommended target levels. Finally, we have used the registry data to look for evidence that treatment improves clinical outcomes.

## 2 Subjects and Methods

### 2.1 Subjects, Ethical Issues and E‐HOD Registry

Information about the E‐HOD registry, patient recruitment and phenotypes has been published previously [2]. The registry was approved by the Ethics Committee of Heidelberg University Hospital (No S‐525/2010; 14 March 2013) and all participating centres received approval from their local ethics committees before enrolling patients. All patients provided written informed consent before pseudonymised data were entered into the registry. In 2017, the participating centres were asked for permission to use the data of the CBS‐deficient patients for analysis and publication, and the project was approved by the E‐HOD Steering Committee. Analysis of data and publication of results was also approved by the Ethics Committee of the General University Hospital in Prague (No 417/20S‐IV).

### 2.2 Definition of Pyridoxine Responsiveness and Dietary Treatment

Degrees of pyridoxine responsiveness were defined and assessed as reported previously [2].

The contributing centres have judged whether each patient was on dietary treatment; we have also reported their intake of natural protein and AAMs.

### 2.3 Data Verification and Inclusion

Data from the registry were extracted on 28 February 2019 and initial verification was undertaken as described previously [2]. Data on treatment relate to what was prescribed and have been retained even if the biochemical results suggest poor compliance. In February 2022, 20 centres were asked to supply missing data or to review outlying data concerning the treatment of 35 patients. Answers were received for all except one patient, for whom the original data were retained (the reported natural protein intake at one visit was low but not impossible at 0.33 g/kg/day with no AAM in a 34‐year‐old).

When data were extracted, the registry held information for 892 visits by 328 patients. Patients and visits were excluded from analysis if essential data (e.g., age and mode of diagnosis) could not be obtained. The reasons for exclusion are shown in Table S1. Emergency visits and those within 3 months of diagnosis were excluded because they did not provide valid information about treatment. The final analyses in this study were based on 834 scheduled visits by 311 patients.

Plasma tHcy and methionine concentrations in 12 patients (28 patient visits) were estimated from values in dried blood spots using published correlation equations [18].

### 2.4 Statistical Analysis

Continuous variables are presented as medians with full range and/or interquartile range (IQR). Categorical variables are shown in absolute and relative frequencies. Differences between responsiveness groups were assessed using Kruskal–Wallis non‐parametric ANOVA for continuous variables and *χ* ^2^ test or Fisher's exact test for categorical variables.

The clinical efficacy of early treatment based on NBS was explored among NRs. Kaplan–Meier time‐to‐event graphs were constructed for lens dislocation event and the first thromboembolic event, comparing the NBS group with (1) the clinically ascertained group censored at the time of diagnosis (i.e., the natural course of the disease) and with (2) this group until the last visit (i.e., the outcome with delayed treatment). Log rank tests were used for the *p* value calculation. Note that clinically ascertained patients were censored at the time of diagnosis if lens dislocation or a thromboembolic event had already occurred because data for the exact timing of the events were not available. The NBS and clinically ascertained groups were also compared using an epidemiological approach, calculating the rate of events in ‘patient years’ of follow‐up (either to the time of diagnosis or until the last visit) and testing for differences using the mid p exact method.

Statistical language and environment R, version 4.3.2 was used throughout the analysis, both for testing and figure creation. The level of statistical significance was set at 0.05.

## 3 Results

### 3.1 Study Population

This study involved 311 subjects with CBS deficiency from the E‐HOD registry. Data on the patients' age and mode of diagnosis, ethnicity, pyridoxine responsiveness and duration of treatment are summarised in Tables S2 and S3. Some patients (13%) were identified by NBS but most were clinically ascertained because of symptoms (selective screening, 71%) or family history (15%). Most patients (61%) were NRs, 20% PRs, 12% FRs and 6% ERs.

### 3.2 Pharmacological and Dietary Treatment Practice

The three main forms of treatment for CBS deficiency are pyridoxine, betaine and a special diet (generally low‐methionine). Table 1 shows the number of patients who were managed with each form of treatment at any stage whilst in the registry, excluding the first 3 months after diagnosis (when patients had trials of pyridoxine responsiveness).

**TABLE 1:** Treatment of patients in the cohort.

		NBS detected	Clinically ascertained (selective and family screening)				
	All	Non and partial responders	Non‐responders	Partial	Full	Extreme	*p*
Number of patients	311	37 + 4	154	59	38	19	—
Pyridoxine, *N*	260 (83.6%)	29 (70.7%)	116 (75.3%)	59 (100.0%)	38 (100.0)	18 (94.7)	< 0.001^a^
Pyridoxine > 10 mg/kg/day, *N*	47	12	26	8	1	0	< 0.001^a^
Pyridoxine dose, median (range), mg/kg/day	3.8 (0.1–34.3)	5.4 (0.2–29.9)	4.3 (0.1–34.3)	4.6 (0.1–14.3)	2.9 (0.6–8.3)	0.7 (0.1–1.9)	< 0.001^b^
Betaine, *N*	204 (65.6%)	29 (70.7%)	132 (85.7%)	42 (71.2%)	1 (2.6%)	0 (0.0%)	< 0.001^a^
Betaine > 200 mg/kg/day, *N*	11	2	8	1	0	—	0.740^a^
Betaine dose, median (range), mg/kg/day	98.3 (4.5–308)	94.1 (18.9–291)	104.5 (4.7–308)	75.8 (4.5–201)	38.3	—	0.053^b^
Special diet, *N*	210 (67.5%)	41 (100.0%)	137 (89.0%)	30 (50.8%)	2 (5.3%)	0 (0.0%)	< 0.001^a^
Natural protein intake, median (range), % WHO safe intake	—	45 (13–305)	53 (8–253)	71(19–153)	—	—	0.149^b^
Amino acid mixtures, *N*	183	39	122	20	2	0	0.010^a^
Intake of amino acid mixtures, median (range), % WHO safe intake	—	110 (45–195)	94 (12–301)	109 (48–172)	—	—	0.169^b^
Total protein intake, median (range), % WHO safe intake	—	157 (74–500)	135 (8–420)	132 (54–253)	—	—	0.288^b^

*Note:* The intake of natural protein, amino acid mixtures and total protein are expressed as a percentage of the age‐appropriate WHO safe protein intake. For drug doses and protein intakes, the mean value has been calculated for each patient, using data for all visits except those within 3 months of diagnosis; the median and range of these mean values have been shown. The natural and total protein intakes were only available for 195 of the 210 patients on dietary treatment and the AAM intake was only available for 179 of the 183 patients on AAMs.

Abbreviation: *N*, number of patients taking the specified form of treatment.

^a^ *p* value of *χ* ^2^ test or Fisher's exact test to check for differences between the groups.

^b^ *p* value of Kruskal–Wallis non‐parametric ANOVA test to test the differences among the groups.

**FIGURE 1:**
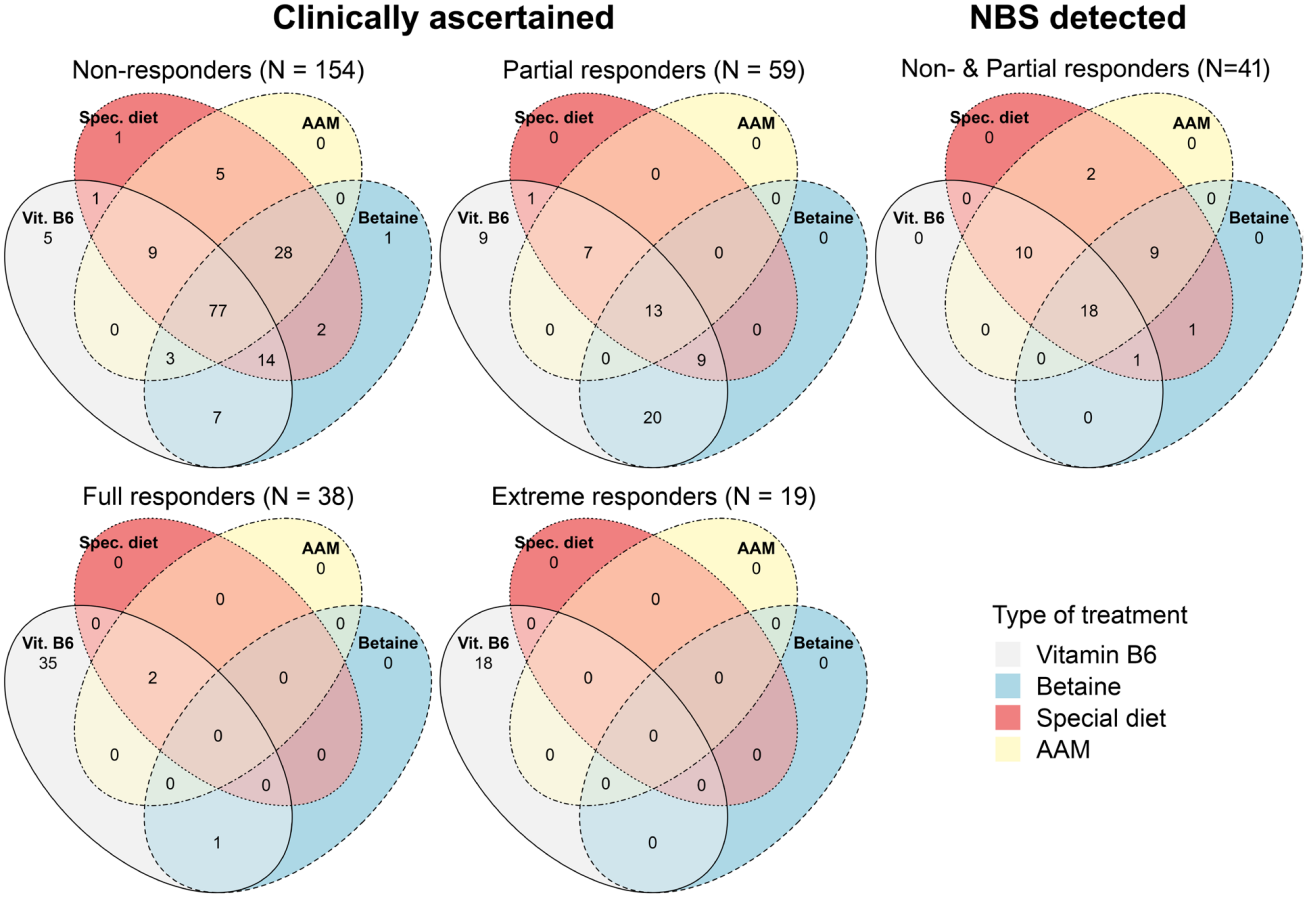
Venn diagrams showing the treatment modalities used in different groups (NBS, NR, PR, FR and ER). One NR and one ER patient were on no treatment when data were entered into the registry (not included in Venn diagrams).

Figure 1 shows the combinations of treatments used in patients detected by NBS and in those ascertained clinically, separated according to their response to pyridoxine. Most NBS, NR and PR patients were on dietary treatment (with or without an AAM) and betaine, whereas very few FRs or ERs were on either. Eight PRs and six NR patients were on neither betaine nor dietary treatment; for seven of the PRs, the tHcy concentrations indicated that they did not need additional treatment; two of the NRs had recently completed trials of pyridoxine responsiveness and one had been lost to follow‐up; it is unclear why the others were not on treatment.

Figure S1 shows the use of diet and betaine in different age groups for patients who are not fully pyridoxine responsive. Almost all the patients aged less than 16 years were on a special diet (with or without betaine) but a number of older patients were on a normal diet. PR patients aged over 32 years were the only group with most patients off dietary treatment.

Table S4 shows other treatments given in addition to pyridoxine, betaine or a special diet. Most patients (85%) were prescribed some form of folate and 47% received some form of vitamin B_12_. Folic acid supplements have been recommended for all patients with CBS deficiency, with B_12_ supplements if patients are deficient [10]. Acetylsalicylic acid was prescribed for 21% of the entire cohort, presumably because they had suffered thromboembolism or were considered at risk. l‐Cystine was prescribed for about a quarter of NR patients and a smaller proportion of PR patients, usually together with an AAM.

### 3.3 Treatment With Pyridoxine

**FIGURE 2:**
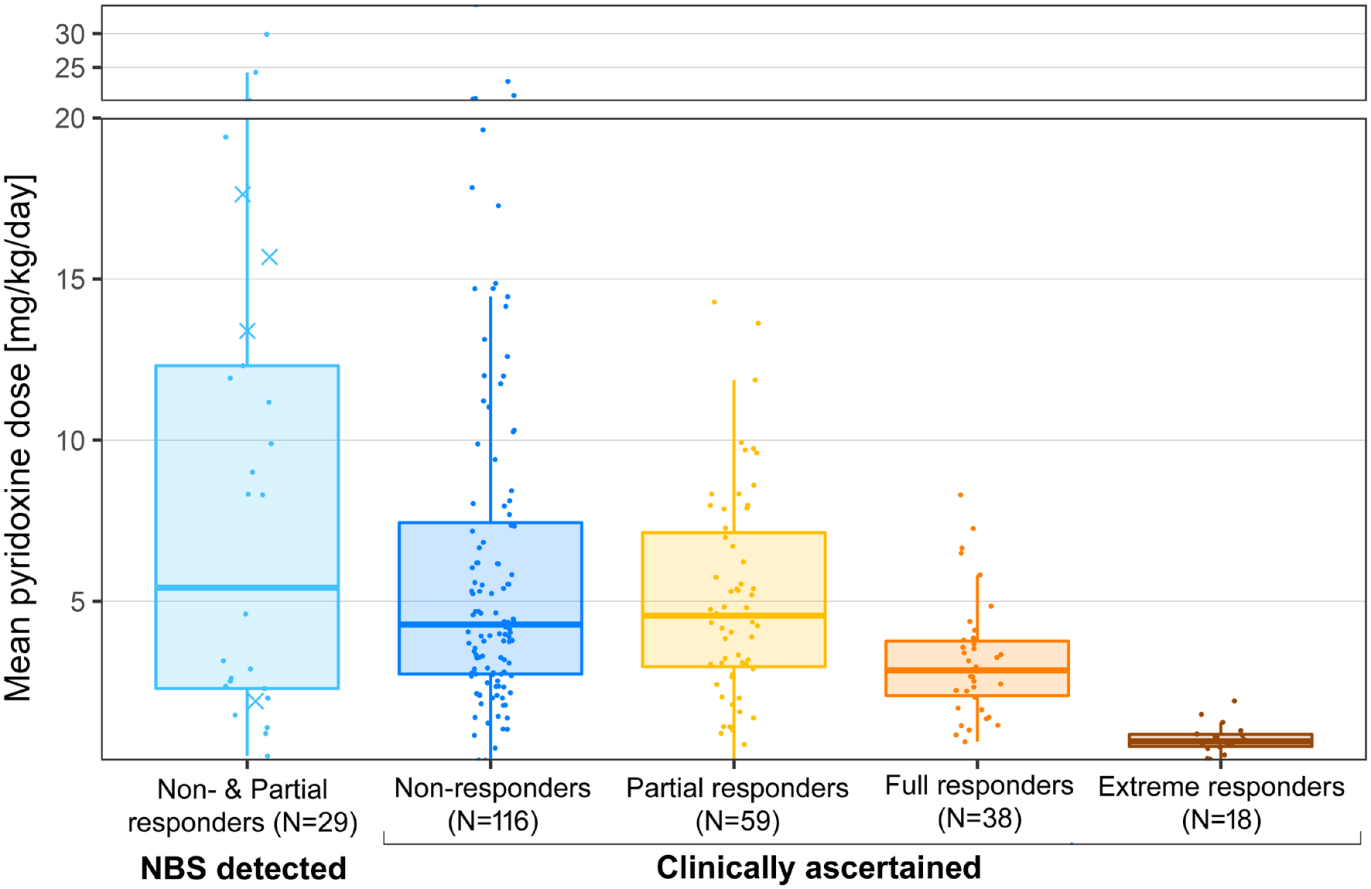
Pyridoxine doses for patients in different groups. For each individual the dose is expressed in mg/kg/day and is the mean of the values at different visits. In the NBS group, partial pyridoxine responders are represented by X and non‐responders by a dot.

Pyridoxine was prescribed for all the patients who showed a response. It was also prescribed for most NR patients. Doses were lowest for ER patients (who were defined as achieving tHcy < 50 μmol/L on < 1 mg/kg/day pyridoxine) and lower for FR patients than for PR or NR patients (Figure 2). Doses above 500 mg/day were prescribed in the long term for 35 patients; a further 13 patients were on doses above 10 mg/kg/day but below 500 mg/day.

### 3.4 Treatment With Betaine

Betaine was prescribed for most NRs and PRs; some of the data have been reported previously [17]. No patients were prescribed doses above 18 g/day, but 11 patients received doses above 200 mg/kg/day; all of these were also on dietary methionine restriction and their highest methionine concentration was 608 μmol/L.

### 3.5 Dietary Treatment

**FIGURE 3:**
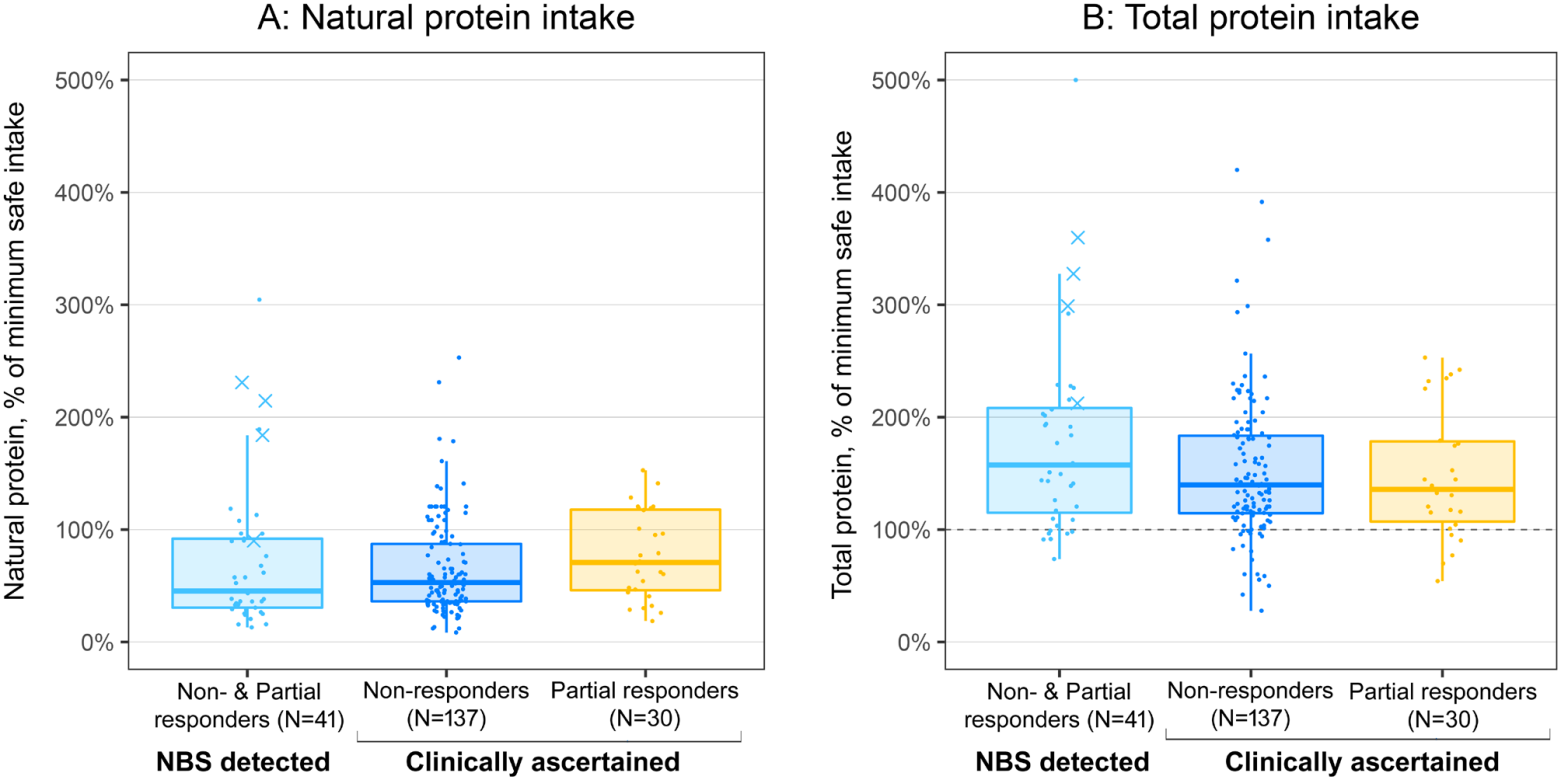
Natural protein consumption and total protein consumption (natural + amino acid mixture) for patients in different groups, expressed as a percentage of the WHO safe protein intake. In the NBS group, partial pyridoxine responders are represented by X and non‐responders by a dot.

Table 1 shows the intake of natural protein, AAM (protein equivalent) and total protein (natural + AAM) for patients treated with a special diet. To compare patients of different ages, the mean protein intake has been calculated for each patient as a percentage of the age‐appropriate WHO (minimum) safe protein intake (Table S5). The data are presented graphically in Figure 3. Some patients were on more natural protein than the WHO safe protein intake but generally still restricted compared to the high‐protein diet of affluent countries. Most patients on dietary treatment were prescribed AAMs, but 41 patients were on diet without an AAM, at least temporarily. For 17 of these, the natural protein intake was above the WHO safe intake and an AAM was subsequently started in 11 of the others. Thirteen patients remained on no AAM despite a protein intake less than the WHO recommendation.

**FIGURE 4:**
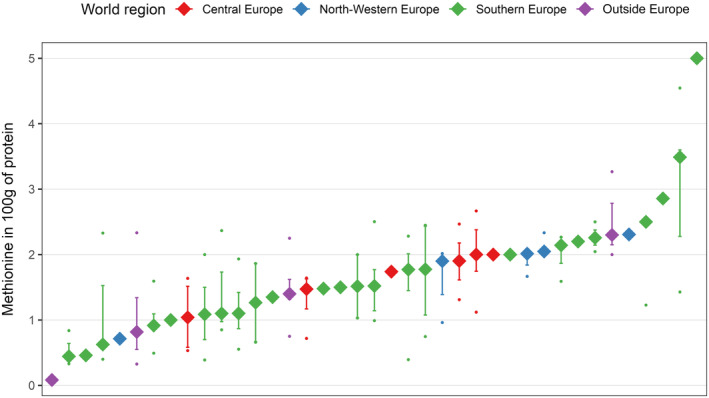
Estimates of the methionine content of 100 g natural protein from different centres' data. These have been calculated when centres provided data for both methionine and natural protein consumption. For each centre, the diamond is the median of the estimates for different patients, the whiskers show the interquartile range and dots show the maximum and minimum. The colour shows the geographical location of the centre.

Figure S2 shows that the proportion of the total protein intake given as AAM varies from 86% to 33% in different centres. For 225 visits, the centres provided estimates of their patients' methionine intake as well as the natural protein intake. We have used these data to calculate the methionine content of 100 g dietary protein and presented the results in Figure 4. There is wide variation within and between the 39 centres that provided these data, unrelated to the geographical location.

### 3.6 Biochemical Results

**FIGURE 5:**
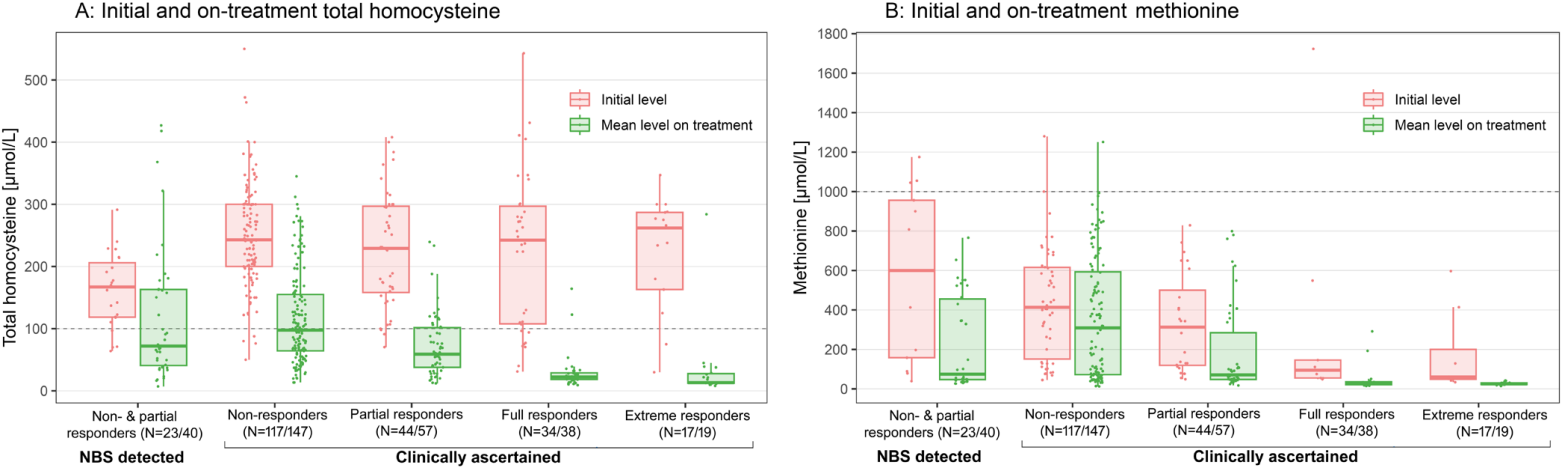
Biochemical efficacy of therapy for patients in different groups. Plasma tHcy and methionine concentrations at diagnosis are compared with the mean values on treatment for each patient whilst enrolled in the registry.

Plasma tHcy concentrations fell with treatment in all groups but to different extents (Table 2 and Figure 5). FRs and ERs had an extremely good response to treatment: all but four patients had a mean plasma tHcy concentration below 50 μmol/L. PRs also showed a good biochemical response: 75% of patients had a mean plasma tHcy concentration below 100 μmol/L. Results were less good for clinically ascertained NRs: almost 50% of patients had a mean plasma tHcy concentration above 100 μmol/L despite treatment, and in 15%, it was above 200 μmol/L. Of the patients detected by NBS, 35% had a mean plasma tHcy concentration above 100 μmol/L on treatment, and in 15%, it was above 200 μmol/L.

**TABLE 2:** Biochemical efficacy of therapy.

		NBS detected	Clinically ascertained				
	All	Non‐ and partial responder	Non‐responder	Partial	Full	Extreme	*p* difference
Number of patients, *N*	311	37 + 4	154	59	38	19	—
Initial total homocysteine, median (range), μmol/L	231.0 (29.9–550)	167.0 (64–291.3)	243.0 (50–550)	229.2 (70.3–408)	242.5 (31–543)	262.0 (29.9–347)	< 0.001^a^
*N* with data for total homocysteine on therapy	301 (96.8%)	40 (97.6%)	147 (95.5%)	57 (96.6%)	38 (100.0%)	19 (100.0%)	0.849^b^
Total homocysteine on therapy,^c^ median (range), μmol/L	68.5 (7–427)	71.9 (7–427)	97.6 (13.5–345)	59.0 (12–239.5)	22.2 (9.2–164.2)	13.3 (8–284)	< 0.001^a^
*N* with total homocysteine > 100 μmol/L at least once	125 (41.5%)	17 (42.5%)	86 (58.5%)	19 (33.3%)	2 (5.3%)	1 (5.3%)	< 0.001^b^
*N* with total homocysteine > 50 μmol/L at least once	—	—	—	—	3 (7.9%)	1 (5.3%)	1.0000^b^
Initial methionine, median (range), μmol/L	349.0 (34–1723)	600.0 (39–1175)	413.5 (45–1280)	313.0 (49–829)	95.0 (49–1723)	60.0 (34–597)	0.006^a^
*N* with data for methionine on therapy	226 (72.7%)	34 (82.9%)	116 (75.3%)	42 (71.2%)	16 (42.1%)	18 (94.7%)	< 0.001^b^
Methionine on treatment,^c^ median (range), μmol/L	94.1 (12.2–1251)	74.2 (26.3–765.9)	308.9 (12.2–1251)	70.8 (17.6–799.2)	29.2 (15–291.5)	24.8 (14.7–42)	< 0.001^a^
*N* with methionine > 1000 μmol/L at least once	9 (4.0%)	0 (0.0%)	7 (6.0%)	2 (4.8%)	0 (0.0%)	0 (0.0%)	0.624^b^

^a^ *p* value of Kruskal–Wallis non‐parametric ANOVA test to test the differences among the groups.

^b^ *p* value of *χ* ^2^ test or Fisher's exact test to check for differences between the groups. *N* = number of patients. The numbers exceeding the specified homocysteine and methionine concentrations relate to patients on treatment. Initial total homocysteine and methionine values were available for 235 and 104 patients, respectively.

^c^ All measurements were in plasma except for 12 patients (28 visits), whose plasma concentrations were estimated from values in dried blood spots. For each patient, the mean of concentrations at different visits has been used; the values shown are the medians and ranges for different patients.

Plasma methionine concentrations also fell with treatment. The median concentration on treatment was 304 μmol/L for the NR group but below 75 μmol/L for all other groups. Methionine concentrations above 600 μmol/L were seen in 50 patients (above 900 μmol/L in 17, of whom 9 were above 1000 μmol/L). All were taking betaine except two children with methionine concentrations of 1015 and 1066 μmol/L, who subsequently started dietary treatment.

### 3.7 Clinical Outcomes

**FIGURE 6:**
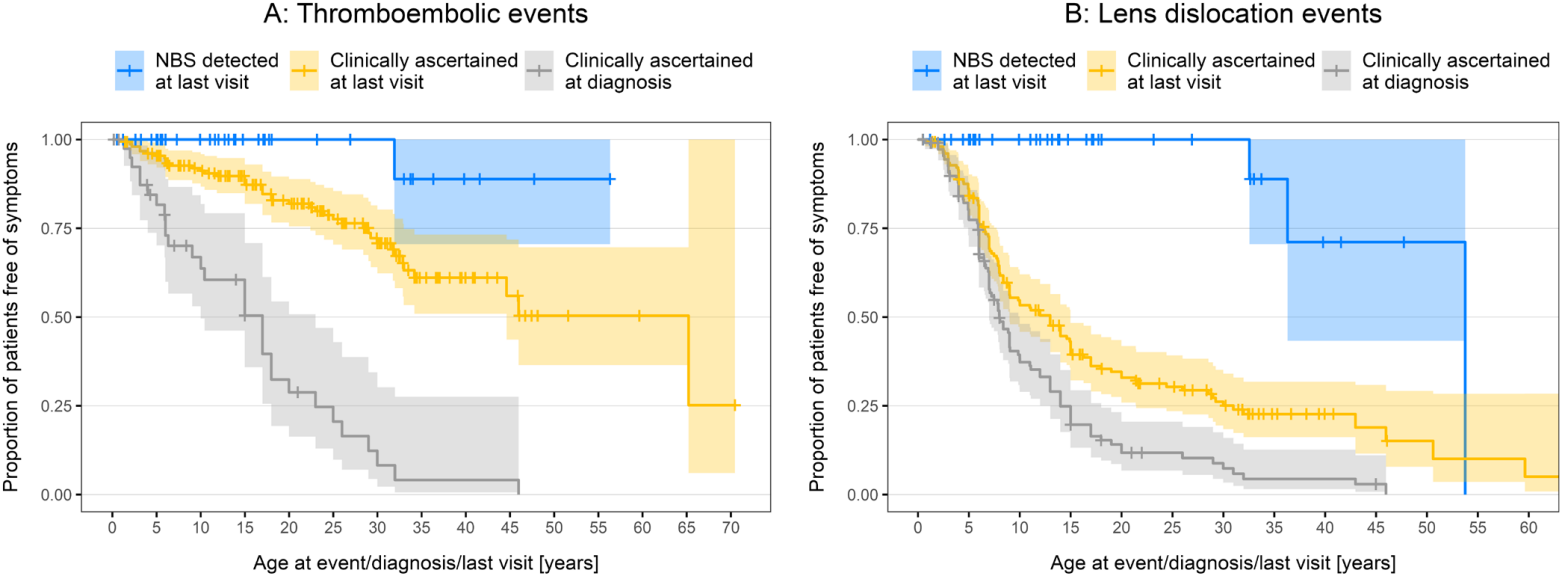
Kaplan–Meier time‐to‐event graphs for first thromboembolic event (Panel A) and lens dislocation (Panel B) in pyridoxine non‐responsive patients. For patients detected by newborn screening, the curve shows the proportion who had not suffered an event at the time of their last visit. For clinically ascertained patients, separate curves show the proportion who had not suffered an event at the time of diagnosis and at the time of their last visit. The shaded areas show 95% confidence intervals. The numbers at risk at 5‐year spaced time points are shown in Table S6.

To assess the clinical efficacy of treatment, time‐to‐event graphs were generated for thromboembolic events and lens dislocation (Figure 6). The 37 NR patients detected by NBS have been compared with the 154 clinically ascertained NRs. For the clinically ascertained group, two curves have been constructed, one showing the proportion of patients who had not suffered the relevant event at the time of diagnosis and one showing the proportion of patients who had not yet suffered the relevant event at the last clinic visit; the first shows the natural history without treatment whereas the second reflects a period on treatment as well as the period of treatment before diagnosis. Both curves are significantly different from the NBS curve for lens dislocation and thromboembolic events. The numbers at risk at 5‐year spaced time points are shown in Table S6. Epidemiological analysis of the data also showed that NBS and early treatment significantly delayed or prevented thromboembolism and lens dislocation when compared with the natural course of the disease or with clinical ascertainment and delayed treatment (Table 3). The relative risk of thromboembolism following NBS was 0.073 compared with the natural course of the disease; for lens dislocation, the relative risk was 0.069 (Table 3).

**TABLE 3:** Statistical analysis of time to thromboembolic event and lens dislocation in pyridoxine non‐responsive patients.

	Number of events	Person years up to the last visit/diagnosis	Event rate	Rate ratio	Rate ratio 95% CI	*p* value (mid *p* exact method)	Survival model: log rank test *p* value
Thromboembolic events							
Efficacy of detection by NBS and early treatment compared to the natural course of the disease							
Clinically ascertained, thromboembolism pre‐diagnosis	30	1433	0.0209	1	—	—	—
NBS detected, up to last visit	1	656	0.0015	0.073	0.010–0.533	< 0.001	< 0.001
Efficacy of detection by NBS and early treatment compared to clinical ascertainment and delayed treatment							
Clinically ascertained, up to last visit	41	3910	0.0105	1	—	—	—
NBS detected, up to last visit	1	656	0.0015	0.145	0.02–1.06	0.013	0.021
Lens dislocation events							
Efficacy of detection by NBS and early treatment compared to the natural course of the disease							
Clinically ascertained, lens dislocation pre‐diagnosis	95	1433	0.0663	1	—	—	—
NBS detected, up to last visit	3	656	0.0046	0.069	0.022–0.218	< 0.001	< 0.001
Efficacy of detection by NBS and early treatment compared to clinical ascertainment and delayed treatment							
Clinically ascertained, up to last visit	111	3910	0.0284	1	—	—	—
NBS detected, up to last visit	3	656	0.0046	0.161	0.051–0.507	< 0.001	< 0.001

*Note:* For both event types, the rate of events in NBS patients until the time of their last visit has been compared (1) with the rate of events in clinically ascertained patients until the time of diagnosis and (2) with the rate of events in clinically ascertained patients until the time of their last visit. The data are from 37 patients detected by NBS and 154 patients ascertained clinically.

Abbreviation: 95% CI, 95% confidence interval.

## 4 Discussion

In 2017, the E‐HOD consortium published evidence‐based guidelines for the diagnosis and management of CBS deficiency [10]. Analysis of data in the registry has shown diverse management that sometimes differs from the guidelines; some data were entered before the guidelines were published and it will be interesting to see if practice changes in the future. In many patients, the mean plasma tHcy concentration was above the recommended target. Nevertheless, the data in the registry show that NBS and treatment from infancy greatly improve outcomes.

### 4.1 Biochemical Efficacy of Treatment

Though the plasma tHcy and methionine concentrations fell with treatment in all groups of patients, the tHcy concentrations fell much lower and more consistently in pyridoxine‐responsive patients. Some NR patients also achieved tHcy values consistently below 100 μmol/L, but many did not. Almost 50% of clinically ascertained NRs and 35% of patients detected by NBS had a mean plasma tHcy concentration above 100 μmol/L, and in 15% of both groups, it was above 200 μmol/L, putting them at high risk of further complications.

Our findings confirm previous observations that betaine treatment alone seldom achieves target tHcy values in NR patients. Of 11 such patients in the registry, 3 had tHcy concentrations of 114, 122 and 129 μmol/L on single visits, but in the others, the mean tHcy ranged from 145 to 312 μmol/L. These 11 patients were aged 24–67 years. All younger NR patients were on dietary treatment (with or without betaine), as recommended in the 2017 guidelines.

Though this study cannot assess compliance with treatment, variation in biochemical control can provide a surrogate marker. For 101 patients, the registry includes tHcy measurements for three or more elective visits whilst on treatment. The tHcy ranges for each patient are presented in Figure S3. The NBS (including mostly NR subjects), NR, PR, FR and ER groups are separated and each patient's treatment is also indicated. For the ERs, the tHcy varies a little. For other groups, the tHcy varies little in some patients but a great deal in others. Intercurrent illnesses can raise tHcy concentrations substantially in NR patients, but they are unlikely to be the explanation as these measurements were all done on elective visits. Issues with compliance are common in many conditions that require long‐term management but they are particularly common in dietary management, which is onerous and has a major impact on lifestyle [19].

### 4.2 Clinical Efficacy of Treatment

Despite imperfect biochemical control, treatment with diet ± betaine greatly reduces the risk of thromboembolism and lens dislocation in NR patients. Figure 6 compares the time to these complications in patients ascertained clinically and by NBS. By the time of analysis, only one of the 37 patients detected by NBS had suffered a thrombosis (aged 32 years) and only two had lens dislocation (aged 32 and 36 years). For clinically ascertained patients, these complications occurred much more frequently and at a younger age. For each complication, the relative risk in the NBS group was 0.07 compared to the risk in the clinically ascertained group censored at diagnosis—that is, the natural course of the disease without treatment; the differences were statistically significant (Table 3). It has not been possible to examine the effect of treatment on other clinical features, such as intelligence and skeletal problems, as fewer of these data have been entered into the registry and there are no clearly defined events.

Few centres undertook NBS for CBS deficiency before 1985. The registry only has data for five NBS patients aged 35 years or more at the time of analysis; these were all from two centres. The conclusions are unchanged, however, even if these patients are excluded.

The difference between the time‐to‐event curves for clinically ascertained patients censored at diagnosis and at their latest visit shows that treatment reduces the risk of thromboembolic events, even if it is not started soon after birth. The difference between the curves is much less for lens dislocation, primarily because this often occurred before treatment was started—it is the commonest symptom leading to diagnosis.

Our results resemble those in previous studies. Yap et al. [20] found far fewer vascular events in a multicentre cohort of 158 treated patients than expected from the classic natural history study of 629 untreated patients [1]; the relative risk was 0.09, very similar to our figure of 0.07. Small, single‐centre studies have shown that treatment from birth can also prevent the other complications of CBS deficiency [20, 21]. Like us, these studies found that treatment from birth can prevent lens dislocation. They also found no intellectual disability or skeletal abnormalities in well‐treated patients; the E‐HOD registry holds insufficient data to address these issues.

### 4.3 Risks of Treatment: Pyridoxine

The main concern about treatment with pyridoxine is that prolonged high doses can cause peripheral neuropathy. The risk is high with doses above 900 mg/day [22, 23], but there are no reliable reports of neuropathy on doses below 500 mg/day [24, 25]. In line with this, the 2017 guidelines recommended using the lowest pyridoxine dose that achieves the biochemical target, up to a maximum of 500 mg/day. Data on the safe pyridoxine dose in children are scarce, but it must be related to size, so we recommended a maximum dose of 10 mg/kg/day [10].

Doses above 10 mg/kg/day were prescribed in the long term for 33 patients in the E‐HOD registry and doses above 500 mg/day for 35 patients. The risk of peripheral neuropathy is low for doses slightly above these values but it is worrying that doses above 900 mg/day were prescribed for 10 patients; if they remain on such doses, they are at high risk of developing neuropathy.

In the past, many NRs were treated with pyridoxine in case they responded in the long term, but there is no evidence that pyridoxine is beneficial in patients who show no biochemical response within 6 weeks. Pyridoxine was prescribed for most NRs in the E‐HOD registry (whether detected clinically or by NBS). This is not a concern if the pyridoxine dose is low, but many NRs were on high doses (Figure 2). Eight of the 10 patients prescribed doses above 900 mg/day were NRs. The pyridoxine will not help these patients but may well cause harm.

### 4.4 Risks of Treatment: Betaine

There is limited guidance on the optimum or maximum safe betaine doses, but the available evidence suggests there is unlikely to be any additional benefit from doses higher than 150–200 mg/kg/day [26, 27]. The manufacturer's current guidance is based partly on data from the E‐HOD registry [17].

The biggest concern with betaine treatment is the possibility of cerebral oedema. This has been reported in 10 CBS‐deficient patients on betaine with methionine concentrations of 972 μmol/L or higher [11]. None of the patients in the E‐HOD registry developed cerebral oedema. Methionine concentrations above 900 μmol/L occurred in 17 patients. Fifteen of these were on treatment with betaine, but only one patient was on a dose above 150 mg/kg/day. Ten were already on dietary treatment, but compliance may have been poor—the methionine concentrations fell without a change in treatment. Betaine doses above 200 mg/kg/day were prescribed for 11 patients in the registry but none had a methionine concentration above 608 μmol/L.

### 4.5 Risks of Treatment: Diet

For dietary treatment, the potential adverse effects are nutritional deficiencies. To achieve target tHcy levels in NR patients, it is usually necessary to restrict natural protein below the age‐appropriate WHO safe protein intake and to give a methionine‐free AAM. Most dietitians aim for a total protein intake above the WHO safe intake, as shown in Figure 3B. This is because the balance of amino acids in the AAMs may not be optimal and the dietary protein is largely from vegetables and has low biological value. Protein‐restricted diets are unlikely to contain enough vitamins and minerals, so the AAMs are generally supplemented with these.

Most patients on dietary treatment were prescribed AAMs, but 13 patients were not on an AAM despite a protein intake that was less than the WHO recommendation. If these patients adhered to the prescribed diet, they were at risk of protein malnutrition and vitamin/mineral deficiencies. The risk of malnutrition is exacerbated by the relatively low biological value of dietary protein in low‐protein diets. Methionine‐free AAMs are unpalatable, and this is probably why they were not taken, but cost and availability may also be factors [19]. The 13 patients were all over 21 years old, which may reflect the difficulty of starting AAMs in older subjects. Other patients could also develop nutritional deficiencies if the AAMs are not taken regularly. The data in the E‐HOD registry do not allow assessment of nutritional status.

A European survey in 2013 found that about half of the metabolic centres based dietary treatment on the protein content of foods and half on its methionine content; most of the latter group, however, used a combination of protein and methionine analysis because data on methionine content are incomplete [28]. Thirty‐nine E‐HOD centres provided data on patients' methionine and protein intake, allowing us to calculate the average methionine content of their dietary protein. Figure 4 shows that the values varied markedly both within and between centres (from 0.08 g methionine/100 g protein to 5.0 g/100 g). Published data indicate that the methionine content of protein ranges from 0.5 g/100 g for fruit to 2.8 g/100 g for milk and 3.2 g/100 g for eggs [29], but patients' diets are unlikely to differ so much, particularly as the methionine/protein ratio was unrelated to the geographical location. The scatter is partly because centres use different methionine/protein ratios for foods whose methionine content is unknown [28] and some centres treat certain fruits and vegetables as methionine‐free and do not count them. The differences are of limited significance, as all centres adjust the diet according to the biochemical results. Nevertheless, harmonisation of dietetic practice might be helpful.

This study has several limitations. Centres entered their own data into the Registry and it has not been possible to verify them independently. Data have not been entered for the interval between diagnosis and recruitment into the Registry; for some patients, data are only available for a small number of visits. The Registry holds few neuropsychology or quality of life data and no imaging or bone densitometry results.

In conclusion, ER and FR patients generally achieved tHcy concentrations below 50 μmol/L. Most NRs were on dietary treatment, usually combined with betaine; betaine alone seldom achieved target tHcy values in NRs. Work is needed on the methionine content of dietary protein, as estimates vary widely. Contrary to the 2017 guidelines, most NR patients were on pyridoxine, sometimes at dangerously high doses. Unfortunately, despite dietary treatment and betaine, many NRs and PRs had a mean tHcy considerably above 100 μmol/L; poor compliance with the onerous diet was probably a major factor. Although committed patients can achieve good results with current management, novel treatments are highly desirable.

## Author Contributions

**Andrew A. M. Morris:** study design, data analysis and interpretation, verification of data quality and consistency, writing of the first draft, manuscript revisions and final approval. **Jitka Sokolová:** verification of data quality and consistency, data management and analysis, preparation of figures and tables of Supporting Information Materials, manuscript revision and final approval. **Markéta Pavlíková:** design and conduction of statistical analyses, interpretation, preparation of figures and tables of Supporting Information Materials, manuscript revision and approval. **Florian Gleich:** participation in study design, concept and maintenance of E‐HOD registry, data verification, manuscript revision and final approval. **Stefan Kölker:** participation in study design, concept and maintenance of the E‐HOD registry, manuscript revision and final approval. **Carlo Dionisi‐Vici:** participation in study design, data interpretation, manuscript revision and final approval. **Matthias R. Baumgartner:** participation in study design, data interpretation, manuscript revision and final approval. **Luciana Hannibal:** participation in data interpretation, manuscript revision and final approval. **Henk J. Blom:** study design, data interpretation, manuscript revision and final approval. **Martina Huemer:** study design, data interpretation, manuscript revision and final approval. **Viktor Kožich:** study design, verification of data quality and consistency, data interpretation, writing of the first draft, manuscript revision and final approval. **E‐HOD Consortium Members:** data acquisition, data entry into registry, verification of data quality and consistency, manuscript revision and final approval.

## Ethics Statement

This study has been carried out in accordance with The Code of Ethics of the World Medical Association (Declaration of Helsinki). The E‐HOD registry was first approved by the Ethics Committee of the University Hospital in Heidelberg (No S‐525/2010; 14 March 2013). All participating centres received approval from their local ethics committees before enrolling patients. Analysis of data and publication of results was also approved by the Ethics Committee of the General University Hospital in Prague (No 417/20S‐IV).

## Consent

All patients provided written informed consent before pseudonymised data were entered into the E‐HOD registry.

## Conflicts of Interest

Andrew A. M. Morris received an honorarium for a lecture from Travere and subsequently provided consultancy to Travere, for which honoraria were paid to E‐HOD funds. Stefan Kölker declares that a Dietmar Hopp Foundation grant was awarded to his institution. Carlo Dionisi‐Vici received consulting fees from Mamoxi and participated in Advisory Board of Nutricia and Vitaflo. Matthias R. Baumgartner declares that a research grant from Nutricia Milupa GmbH was awarded to his institution. Martina Huemer declares consultancy honoraria or travel support from Nutricia Metabolics, Sanofi, Chiesi and Immedica Pharma, all unrelated to this work. Her institution has received unrestricted research grants from Nutricia Metabolics, Sanofi and Travere. Viktor Kožich provided consultancy to GAIN Therapeutic and Travere, honoraria were paid to E‐HOD funds. The other authors declare no conflicts of interest. E‐HOD has been receiving support for its activities from Aeglea, Gain Therapeutic, Nutricia Metabolics, Recordati, SOBI, Travere and Vitaflo. The authors confirm independence from the sponsors; the content of the article has not been influenced by the sponsors.

## Supporting information


**Data S1.** Supporting Information.

## Data Availability

The data that support the findings of this study are available on request from the corresponding authors. The data are not publicly available due to privacy or ethical restrictions.

## Appendix A

E‐HOD Consortium Members

Rodrigo R. Arantes, Francisco Arrieta Blanco, Anna Baghdasaryan, Diana Ballhausen, Javier Blasco‐Alonso, Martijn Brouwers, María Bueno, Rosa Burgos, Elvira Cañedo Villarroya, Aline Cano, María‐Luz Couce, Ellen Crushell, Javier De Las Heras, Can Ficicioglu, María Concepción García Jiménez, Ana Gaspar, Domingo González‐Lamuño Leguina, Kimberly A. Chapman, Yin‐Hsiu Chien, Mirian C. H. Janssen, Pavel Ješina, Christina Kaufman, Robin Lachmann, Christian Lavigne, Allan M. Lund, Natalia Lüsebrink, Francois Maillot, Ana Maria Martins, Silvia Meavilla Olivas, Karine Mention, Isidro Vitoria Miñana, Fanny Mochel, Ahmad Monavari, Sónia Moreira, Carolina Araujo Moreno, Helen Mundy, Elaine Murphy, Giorgia Olivieri, Stéphanie Paquay, Luís Peña‐Quintana, Danijela Petković Ramadža, Gloria Liliana Porras‐Hurtado, Pilar Quijada‐Fraile, Isabelle Redonnet‐Vernhet, Alexander Rennings, Mònica Ruiz Pons, Saikat Santra, Aude Servais, Maria Cristina Schiaffino, Manuel Schiff, Bernd C. Schwahn, Ida V. D. Schwartz, Leighann J. Sremba, Collette Stainforth, Karolina Maria Stepien, Jolanta Sykut‐Cegielska, Allyson Terry, Inmaculada Vives Piñera, Monique Williams, Jiří Zeman, Matthias Zielonka

Affiliations of E‐HOD consortium members.

^1^Pediatrics, Universidade Federal de Minas Gerais (UFMG), Belo Horizonte, Brazil

^2^Endocrinology & Nutrition, Hospital Universitario Rey Juan Carlos, Madrid, Spain

^3^Metabolic congenital disease (CSUR‐MetabERN), Hospital Universitario Ramón y Cajal, Madrid, Spain

^4^Department of Pediatrics and Adolescent Medicine, Division of General Pediatrics, Medical University of Graz, Graz, Austria

^5^Pediatric Metabolic Unit, Pediatrics, Woman‐Mother–Child Department, University of Lausanne and University Hospital of Lausanne, Lausanne, Switzerland

^6^Pediatric Gastroenterology and Nutrition Unit, Hospital Regional Universitario de Málaga, Malaga, Spain

^7^Internal Medicine, Division of Endocrinology and Metabolic Disease, Maastricht University Medical Centre, Maastricht, Netherlands

^8^Paediatric Metabolic Diseases, Hospital Universitario Virgen del Rocío, Sevilla, Spain

^9^Nutritional Support Unit, University Hospital Vall d'Hebron, Barcelona, Spain

^10^Section of Pediatric Gastroenterology and Nutrition, Hospital Infantil Universitario Niño Jesús, Madrid, Spain

^11^Reference Center of Inherited Metabolic Disorders, La Timone Enfant Hospital, Marseille, France

^12^Metabolic Unit, University Clinical Hospital of Santiago de Compostela, Santiago de Compostela, Spain

^13^National Centre for Inherited Metabolic Disorders, Children's Health Ireland, Temple St, Dublin and University College Dublin, Dublin, Ireland

^14^Department of Pediatrics, Hospital Universitario Cruces, Bilbao, Spain

^15^Department of Pediatrics, University of the Basque Country, Bilbao, Spain

^16^Department of Inborn Errors of the Metabolism, BioBizkaia Health Research Institute, Bilbao, Spain

^17^Division of Human Genetics, The Children's Hospital of Philadelphia, Perelman School of Medicine at the University of Pennsylvania, Philadelphia, PA, USA

^18^Metabolic Department, University Children Miguel Servet Hospital, IIS Aragon, Zaragoza, Spain

^19^Pediatrics, Unidade Local de Saúde Santa Maria (ULSSM), Lisbon, Portugal

^20^Department of Pediatrics, University Hospital Marques de Valdecilla‐Universidad de Cantabria, Santander, Spain

^21^Children's National Hospital, Washington DC, USA

^22^Department of Medical Genetics, National Taiwan University Hospital, Taipei, Taiwan

^23^Internal Medicine, Radboud University Medical Centre, Nijmegen, Netherlands

^24^Department of Pediatrics and Inherited Metabolic Disorders, Charles University‐First Faculty of Medicine and General University Hospital in Prague, Prague, Czechia

^25^Division of Metabolism and Children's Research Center, University Children's Hospital Zurich, Zurich, Switzerland

^26^Charles Dent Metabolic Unit, National Hospital for Neurology and Neurosurgery, London, UK

^27^Internal Medicine and Clinical Immunology Department, University Hospital of Angers, Angers, France

^28^Centre for Inherited Metabolic Diseases, Departments of Clinical Genetics and Paediatrics, Copenhagen University Hospital, Copenhagen, Denmark

^29^Department of Pediatrics, Goethe University Frankfurt, Frankfurt am Main, Germany

^30^Internal Medicine, University Hospital of Tours, Tours, France

^31^Pediatrics, Universidade Federal de São Paulo, São Paulo, Brazil

^32^Pediatric, Gastroenterology, Hepatology and Nutrition, Hospital Sant Joan de Déu, Barcelona, Spain

^33^Centre de référence des Maladies Héréditaires du métabolisme, Hôpital Jeanne de Flandre, Lille, France

^34^Unit of Metabolopathies, Universitary Hospital La Fe, Valencia, Spain

^35^Reference Center for Adult Neurometabolic disorders, Department of Genetics, La Pitie‐Salpetriere University Hospital, Paris, France

^36^Internal Medicine, Centro Hospitalar e Universitário de Coimbra, Coimbra, Portugal

^37^Medical Genetics, State University of Campinas, Campinas, Brazil

^38^Evelina London Children's Hospital, London, UK

^39^Division of Metabolic Diseases and Hepatology, Bambino Gesù Children's Hospital, IRCCS, Rome, Italy

^40^Pediatric Neurology and Inborn Errors of Metabolism, Université Catholique de Louvain, Cliniques Universitaires Saint‐Luc, Bruxelles, Belgium

^41^Clinical Sciences, University of Las Palmas de Gran Canaria, Las Palmas de Gran Canaria, Spain

^42^Pediatric Gastroenterology, Hepatology and Nutrition Unit, Mother and Child Insular University Hospital Complex, CIBEROBN ISCIII, Las Palmas de Gran Canaria, Spain

^43^Department of Pediatrics, University Hospital Center Zagreb and University of Zagreb, School of Medicine, Zagreb, Croatia

^44^Research Unit Department, Orphan Disease Line, Institution Comfamiliar Risaralda, Pereira, Colombia

^45^Pediatrics, Hospital Universitario 12 de Octubre, Madrid, Spain

^46^Endocrinologie‐Nutrition‐Maladies Métaboliques Hopital Haut‐Lévêque, CHU Bordeaux, Bordeaux, France

^47^Department of Pediatrics, Hospital Universitario Nuestra Señora de la Candelaria, Santa Cruz de Tenerife, Spain

^48^Department of Clinical Inherited Metabolic Disorders, Birmingham Women's and Children's NHS Foundation Trust, Birmingham, UK

^49^Nephrology and Transplantation, MAMEA Reference Center, Necker Hospital, APHP, Paris, France

^50^Pediatric Clinic and Endocrinology Unit, IRCCS Instituto Giannina Gaslini, Genova, Italy

^51^Necker Hospital, APHP, Reference Center for Inborn Error of Metabolism, Pediatrics Department, University of Paris, Paris, France

^52^Inserm UMR_S1163, Institut Imagine, Paris, France

^53^Manchester Centre for Genomic Medicine, St Mary's Hospital, Manchester University NHS Foundation Trust, Health Innovation Manchester, Manchester, UK

^54^Division of Evolution & Genomic Sciences, School of Biological Sciences, Faculty of Biology, Medicine and Health, University of Manchester, Manchester, UK

^55^Medical Genetics Service and Department of Genetics, Hospital de Clínicas de Porto Alegre and Universidade Federal do Rio Grande do Sul, Porto Alegre, Brazil

^56^Department of Pediatrics, University of Colorado, Aurora, USA

^57^Bradford Teaching Hospitals NHS Foundation Trust, Bradford, UK

^58^Adult Inherited Metabolic Diseases, Salford Royal Organisation, Northern Care Alliance NHS Foundation Trust, Salford, UK

^59^Department of Inborn Errors of Metabolism and Pediatrics, The Institute of Mother and Child, Warsaw, Poland

^60^Dietetic Department, Alder Hey Children's NHS Foundation Trust, Liverpool, UK

^61^Department of Inherited Metabolic Disorders and Pediatrics, Hospital Universitario Virgen de la Arrixaca, Murcia, Spain

^62^Department of Pediatrics, Center for Lysosomal and Metabolic Diseases, Erasmus MC—Sophia Children's Hospital, University Medical Center Rotterdam, Rotterdam, Netherlands

^63^Centre for Pediatric and Adolescent Medicine, Department of Pediatrics I, Division of Pediatric Neurology and Metabolic Medicine, Heidelberg University, Medical Faculty of Heidelberg, Heidelberg, Germany
