# Supplementary material for: Cystathionine β‐Synthase Deficiency in the E‐HOD Registry—Part II: Dietary and Pharmacological Treatment
Source: J Inherit Metab Dis. 2025 Jan 13;48(1):e12844. doi: 10.1002/jimd.12844 (PMC11729643; doi:10.1002/jimd.12844)
Supplement: Supplementary file 1 — Data S1. Supporting Information. [file JIMD-48-0-s001.pdf]

Supplementary materials

Supplementary Table S1: Data in the E-HOD registry excluded from analysis....2

Supplementary Table S2. Ascertainment and pyridoxine responsivity of patients 2

Supplementary Table S3. Characteristics of the cohort....3

Supplementary Table S4: Additional treatments (besides pyridoxine, betaine and diet) 4

Supplementary Table S5: WHO requirements used to normalize protein and/or methionine intake....5

Supplementary Table S6: Numbers at risk in the time-to-event graphs (Figure 6) at 5-year spaced time-points....6

Supplementary Figure S1: Treatment modalities used in patients of different ages within the newborn screening, pyridoxine non-responder and partial responder groups. 7

Supplementary Figure S2: Proportion of total protein intake given as amino acid mixture....8

Supplementary Figure S3: Range of plasma total homocysteine concentrations in each patient....9

Supplementary Table S1: Data in the E-HOD registry excluded from analysis

Reason for exclusion	Patients	Visits
	N=328	N=892
Missing age at diagnosis	7	7
Missing mode of diagnosis	6	6
Only data within 3 months of diagnosis	4	5
Emergency visit		10 (from 7 patients)
Fatal visit		1
Missing weight, therapy and tHcy		25 (from 19 patients)
Data on treatment in patients within 3 months since diagnosis		4 (from 4 patients)
	N=311	N=834

Supplementary Table S2. Ascertainment and pyridoxine responsivity of patients

	**All**		**Non-responder**		**Partial**		**Full**		**Extreme**	
	N	*%*	*N*	*%*	*N*	*%*	*N*	*%*	*N*	*%*
**Selective screening**	222	71.4	127	66.5	52	82.5	29	76.3	14	73.7
**Family screening**	48	15.4	27	14.1	7	11.1	9	23.7	5	26.3
**Newborn screening**	41	13.2	37	19.4	4	6.3	0	0.0	0	0.0
**All**	311	100.0	191	100.0	63	100.0	38	100.0	19	100.0

Supplementary Table S3. Characteristics of the cohort

		NBS detected	Clinically ascertained (selective & family screening)				
	**All**	**Non-responders and partial responders**	**Non-responders**	**Partial**	**Full**	**Extreme**	***p difference***
**Number of patients**	311	37+4	154	59	38	19	–
**Males,** N (%)	168 (54.0%)	23 (56.1%)	89 (57.8%)	36 (61.0%)	15 (39.5%)	5 (26.3%)	*0.025*[1]
**Females,** N (%)	143 (46.0%)	18 (43.9%)	65 (42.2%)	23 (39.0%)	23 (60.5%)	14 (73.7%)	
**White,** N (%)	278 (92.4%)	40 (97.6%)	137 (91.3%)	49 (87.5%)	35 (94.6%)	17 (100.0%)	*0.330*[1]
**Other,** N (%)	23 (7.6%)	1 (2.4%)	13 (8.7%)	7 (12.5%)	2 (5.4%)	0 (0.0%)	
**Age at diagnosis** median (range), *years*	7.0 (0 - 73)	0.03 (0 - 0.3)	6.9 (0 - 46)	11.0 (0 - 55)	21.5 (0.1 - 62)	33.0 (0 - 73)	*< 0.001*[2]
**Patient years prior diagnosis**, *years*	3883	2	1433	897	910	641	–
**Duration of treatment** median (range), *years*	13.2 (0.3 - 56.3)	12.4 (0.5 - 56.3)	14.2 (0.3 - 50.6)	17.3 (0.3 - 52)	10.8 (0.7 - 41.1)	4.9 (0.3 - 55.1)	*0.059*[2]
**Patient years on treatment**, *years*	5045	672.3	2477.4	1103.8	589.6	201.5	–
**Visits per patient** median (range),	2 (1 - 12)	2 (1 - 6)	2 (1 - 12)	2 (1 - 12)	1 (1 - 9)	3 (1 - 10)	*0.006*[2]
**Total visits**	834	94	437	177	72	54	–

[1]p-value of χ^2-test to analyse differences between the groups in distribution of gender/race

[2]p-value of Kruskall-Wallis non-parametric ANOVA test for differences among the responsivity groups

N = number of patients. Valid N = number for whom the information was available.

Supplementary Table S4: Additional treatments (besides pyridoxine, betaine and diet)

		NBS detected	Clinically ascertained (selective screening and family screening)			
	**All**	**Non- and partial responders**	**Non-responders**	**Partial**	**Full**	**Extreme**
**Number of patients,** N	311	37+4	154	59	38	19
**Any form of vitamin B12,** N	146 (47%)	18 (44%)	77 (50%)	29 (49%)	13 (34%)	9 (47%)
**Hydroxycobalamin,** N	76 (24%)	13 (32%)	39 (25%)	15 (25%)	3 (7.9 %)	6 (32%)
**Cyanocobalamin,** N	90 (29%)	7 (17%)	51 (33%)	18 (31%)	11 (29%)	3 (16%)
**Methylcobalamin,** N	3 (1.0%)	1 (2.4%)	1 (0.6%)	0 (0%)	0 (0%)	1 (5.3%)
**Any form of folate,** N	263 (85%)	31 (76%)	133 (86%)	56 (95%)	29 (76%)	14 (74%)
**Folic acid,** N	246 (79%)	30 (73%)	121 (79%)	53 (90%)	29 (76%)	13 (68%)
**Folinic acid,** N	29 (9.3%)	1 (2.4%)	17 (11%)	7 (12%)	1 (2.6%)	3 (16%)
**5-methylfolic acid,** N	2 (0.6%)	1 (2.4%)	1 (0.6%)	0 (0%)	0 (0%)	0 (0%)
**L-carnitine,** N	14 (4.5%)	1 (2.4%)	8 (5.2%)	4 (6.8%)	1 (2.6%)	0 (0%)
**Ascorbic acid,** N	19 (6.1%)	3 (7.3%)	10 (6.5%)	4 (6.8%)	0 (0%)	2 (11%)
**Acetylsalicylic acid,** N	66 (21%)	2 (4.9%)	40 (26%)	18 (31%)	2 (5.3%)	4 (21%)
**L-cystine,** N	63 (20%)	9 (22%)	44 (29%)	8 (14%)	2 (5.3%)	0 (0%)
**N-acetylcysteine**	1 (0.3%)	1 (2.4%)	0 (0%)	0 (0%)	0 (0%)	0 (0%)

N = number of patients taking the specified form of treatment.

Supplementary Table S5: WHO requirements used to normalize protein and/or methionine intake

**Age**, *years*	**Safe protein intake**, *g/kg/d*		**Sulfur amino acid requirements**, *mg/kg/d*	**Estimated methionine requirements**, *mg/kg/d*
	**M**	**F**		
0 - 0.75	1.31	1.31	31	22.2
0.75 - 1.25	1.14	1.14	22	15.8
1.25 - 1.75	1.03	1.03	22	15.8
1.75 – 2.5	0.97	0.97	22	15.8
2.5 – 3.5	0.9	0.9	18	12.9
3.5 – 6.5	0.87	0.87	18	12.9
6.5 – 10.5	0.92	0.92	18	12.9
10.5 – 14.5	0.9	0.89	17	12.2
14.5 - 18	0.87	0.84	16	11.5
>18	0.83	0.83	15	10.8

Data from WHO Technical Report Series 935: Protein and amino acid requirements in human nutrition (2007). Data on protein and sulfur amino acid intake are on p244 and p246, respectively. Gaps between the age categories in the WHO reference have been divided equally. For example, the WHO requirements for age 3 years were considered appropriate for age 2.5 to 3.5 years and those for age 4-6 years were considered appropriate for age 3.5-6.5 years. The methionine requirement was estimated as 67% of the sulfur amino acid requirement (the mean proportion in liver and muscle protein, p145 of WHO report).

Supplementary Table S6: Numbers at risk in the time-to-event graphs (Figure 6) at 5-year spaced time-points

**TE**	Age [years]	0	5	10	15	20	25	30	35	40	45	50	55	60	65	70	75
	NBS at last visit	37	32	24	16	11	10	9	5	3	2	1	1	0	0	0	0
	Clinically ascertained at last visit	154	143	126	106	88	67	47	25	16	11	4	3	2	2	1	0
	Clinically ascertained, before treated	41	29	21	14	6	5	2	1	1	1	0	0	0	0	0	0
**LD**	Age [years]	0	5	10	15	20	25	30	35	40	45	50	55	60			
	NBS at last visit	37	32	24	16	11	10	9	5	3	2	1	0	0			
	Clinically ascertained at last visit	154	127	77	53	44	33	23	12	7	5	3	2	0			
	Clinically ascertained, before treated	111	81	37	18	10	8	5	3	3	1	0	0	0			

TE = thromboembolism, LD = lens dislocation

Supplementary Figure S1: Treatment modalities used in patients of different ages within the newborn screening, pyridoxine non-responder and partial responder groups.

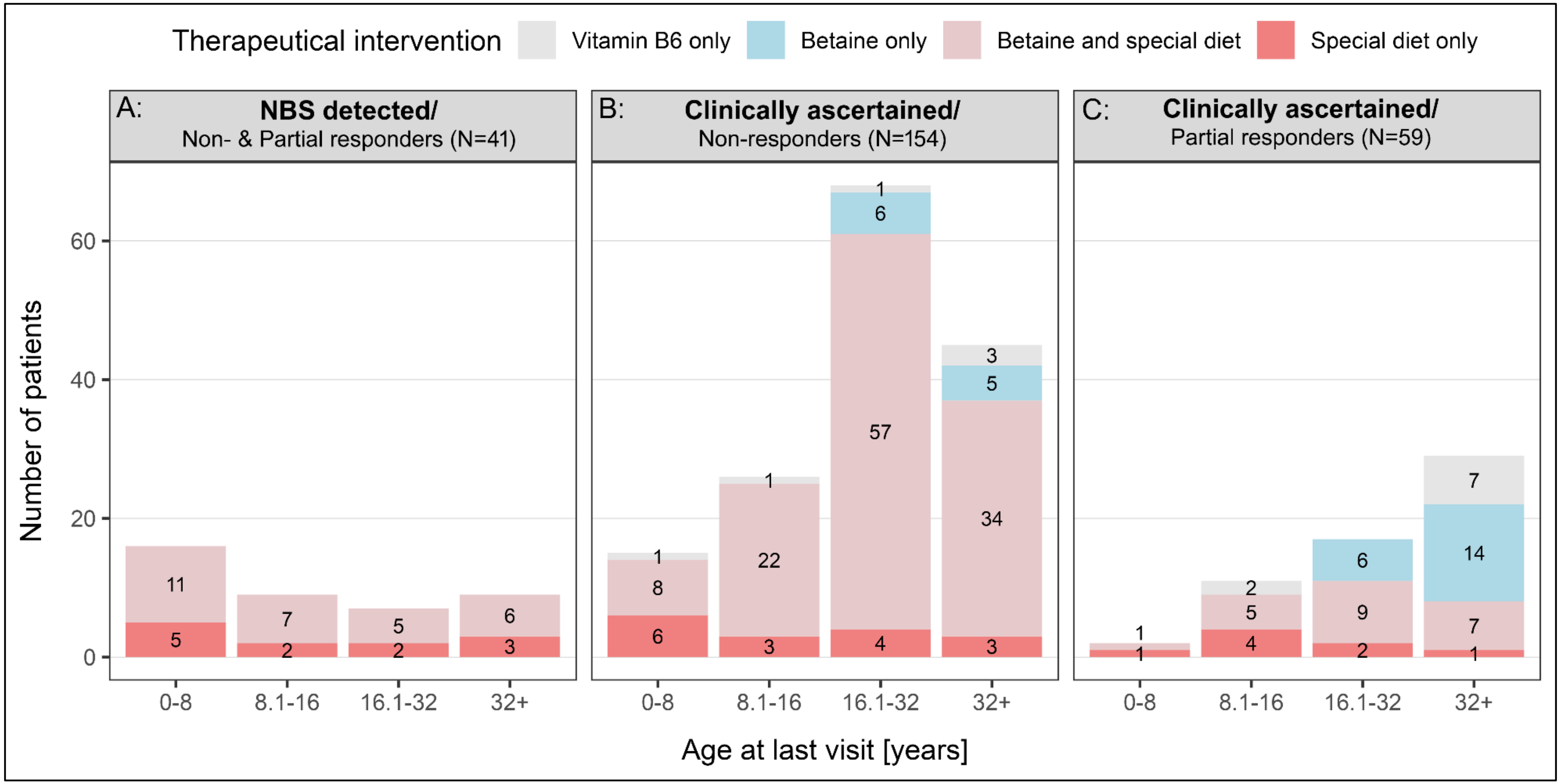

Supplementary Figure S1: Treatment modalities used in patients of different ages in the NBS, NR and PR groups. Treatment is included if given at any stage whilst the patient was enrolled in the registry. Treatment with pyridoxine is only indicated for patients on neither betaine nor diet. The age is that at the last visit.

Supplementary Figure S2: Proportion of total protein intake given as amino acid mixture

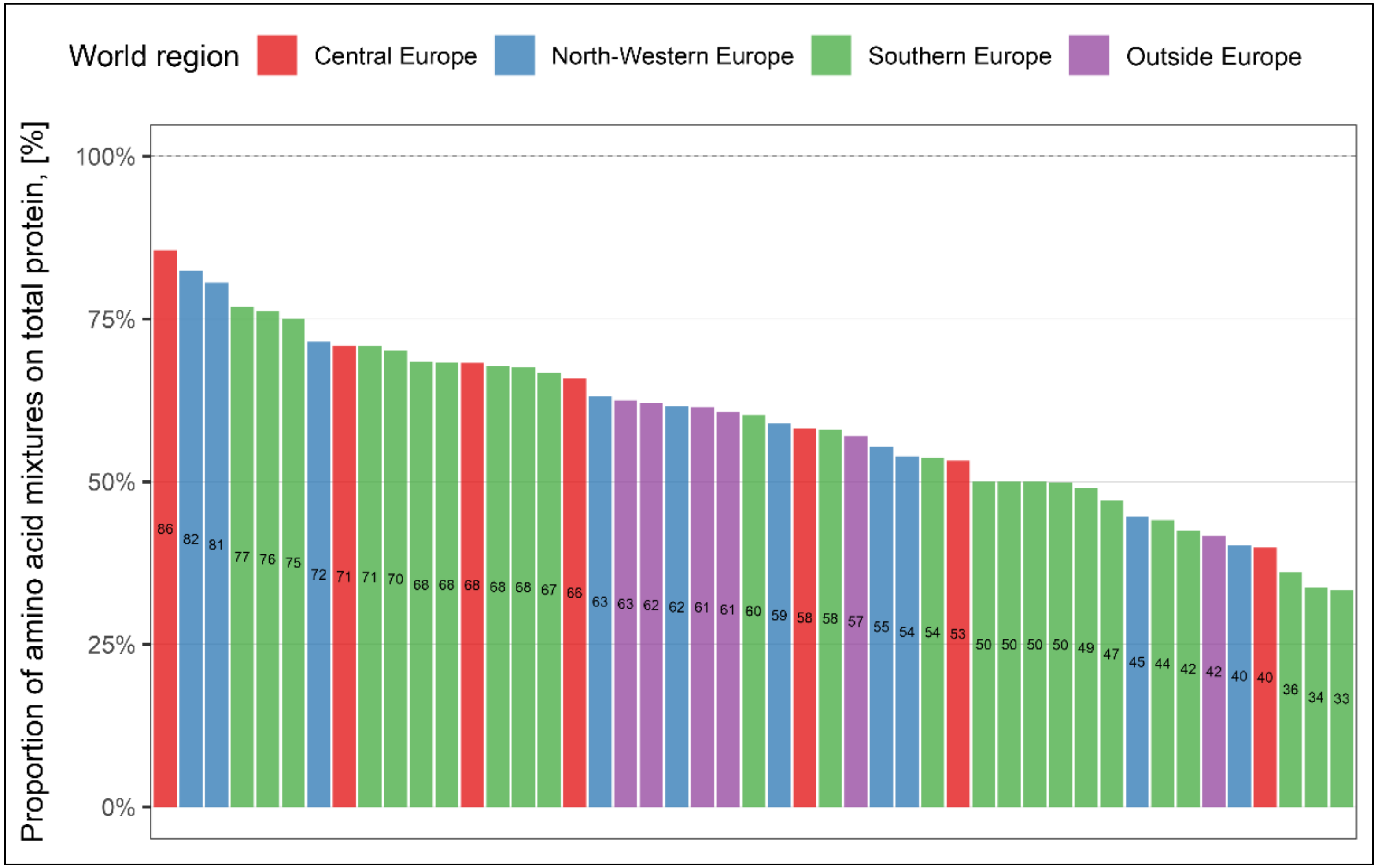

Supplementary Figure S2: *Proportion of the total protein intake given as an amino acid mixture. Each value is the mean for patients on dietary management at one centre. The colour shows the geographical location of the centre.*

Supplementary Figure S3: Range of plasma total homocysteine concentrations in each patient

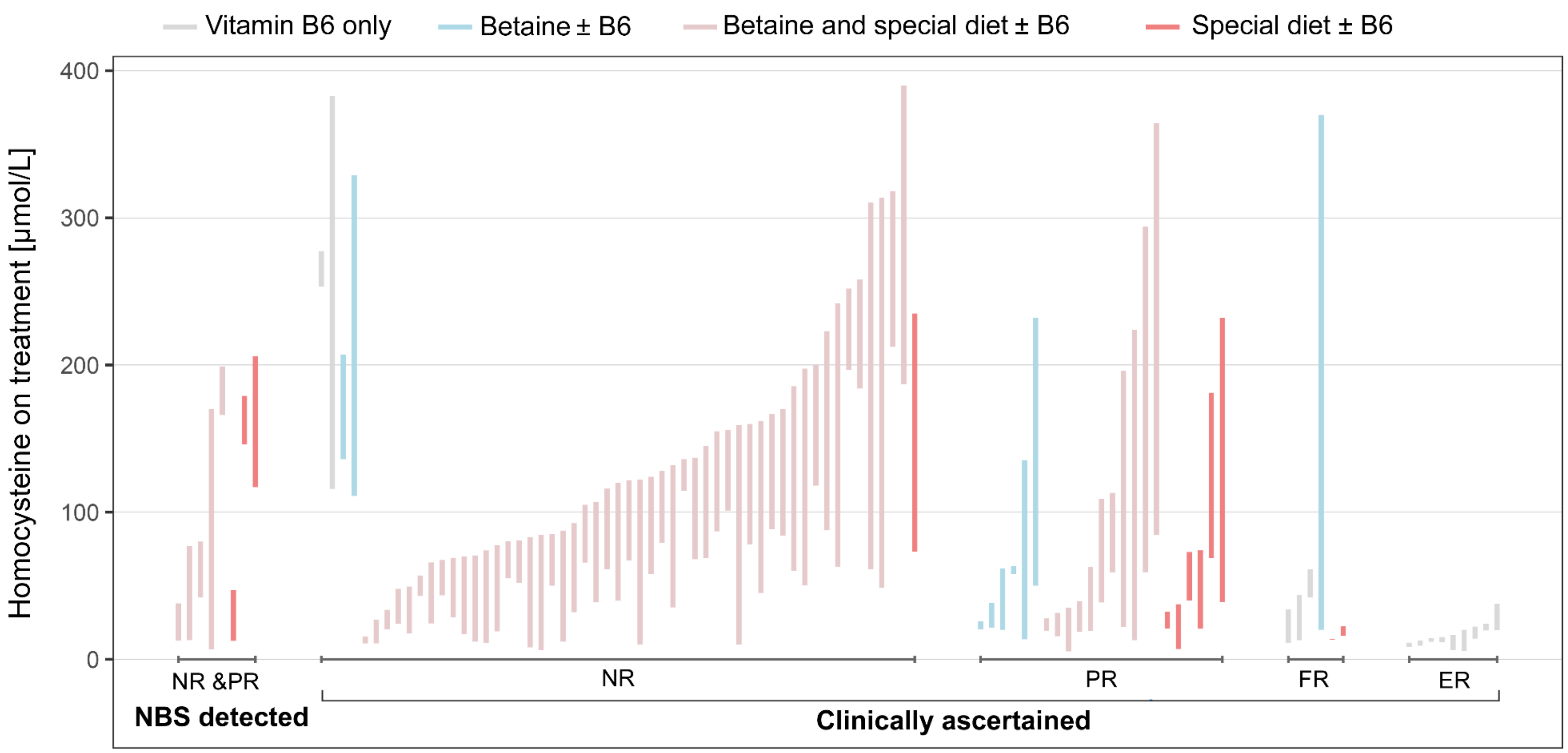

Supplementary Figure S3: *Range of plasma total homocysteine concentrations in each of the 101 patients with values for at least three visits. Patients have been arranged by forms of treatment and maximum homocysteine value.*
